# Increased Bone Mass in Female Mice Lacking Mast Cell Chymase

**DOI:** 10.1371/journal.pone.0167964

**Published:** 2016-12-09

**Authors:** Thomas Lind, Ann-Marie Gustafson, Gabriela Calounova, Lijuan Hu, Annica Rasmusson, Kenneth B. Jonsson, Sara Wernersson, Magnus Åbrink, Göran Andersson, Sune Larsson, Håkan Melhus, Gunnar Pejler

**Affiliations:** 1 Uppsala University Hospital, Department of Medical Sciences, Section of Clinical Pharmacology, Uppsala, Sweden; 2 Uppsala University, Department of Medical Biochemistry and Microbiology, Uppsala, Sweden; 3 Uppsala University Hospital, Department of Surgical Sciences, Uppsala, Sweden; 4 Swedish University of Agricultural Sciences, Department of Anatomy, Physiology and Biochemistry, Uppsala, Sweden; 5 Swedish University of Agricultural Sciences, Department of Biomedical Science and Veterinary Public Health, Uppsala, Sweden; 6 Karolinska Institute, Division of Pathology, Department of Laboratory Medicine, Karolinska University Hospital Huddinge, Stockholm, Sweden; Universite de Nantes, FRANCE

## Abstract

Here we addressed the potential impact of chymase, a mast-cell restricted protease, on mouse bone phenotype. We show that female mice lacking the chymase Mcpt4 acquired a persistent expansion of diaphyseal bone in comparison with wild type controls, reaching a 15% larger diaphyseal cross sectional area at 12 months of age. *Mcpt4*^*-/-*^ mice also showed increased levels of a bone anabolic serum marker and higher periosteal bone formation rate. However, they were not protected from experimental osteoporosis, suggesting that chymase regulates normal bone homeostasis rather than the course of osteoporosis. Further, the absence of Mcpt4 resulted in age-dependent upregulation of numerous genes important for bone formation but no effects on osteoclast activity. In spite of the latter, *Mcpt4*^*-/-*^ bones had increased cortical porosity and reduced endocortical mineralization. Mast cells were found periosteally and, notably, bone-proximal mast cells in *Mcpt4*^*-/-*^ mice were degranulated to a larger extent than in wild type mice. Hence, chymase regulates degranulation of bone mast cells, which could affect the release of mast cell-derived factors influencing bone remodelling. Together, these findings reveal a functional impact of mast cell chymase on bone. Further studies exploring the possibility of using chymase inhibitors as a strategy to increase bone volume may be warranted.

## Introduction

Fractures of the skeleton, especially at the hip, represent devastating injuries, resulting in disability, increased mortality and high treatment costs. The risk of hip fractures increases with age and, as a consequence of the aging human population, the incidence of fractures is expected to increase within the society [1]. The effectiveness of current treatments available to prevent fractures are low and, hence, there is a large need for identifying novel mechanisms operative in bone homeostasis (especially those promoting bone growth), thereby forming the basis for developing better ways to improve bone strength [2,3].

Mast cells are present in most tissues, placed around blood vessels and nerves, and are especially prominent at host-environment interfaces, such as skin, lungs, digestive tract, nose, eyes and ears. Mast cells are well-known for their detrimental impact in allergic disorders, but there is a rising awareness of a role for mast cells in various additional pathologies, including, e.g., arthritis, atherosclerosis, cancer and obesity [4–6]. However, the exact mechanisms by which mast cells participate in these diseases are in many cases unclear. A hallmark feature of mast cells is their large content of secretory granules, filled with high amounts of various preformed compounds, including monoamines such as histamine and serotonin, certain cytokines (e.g. TNF), serglycin proteoglycans and a variety of mast cell-specific proteases [7], the latter encompassing serine proteases of tryptase- or chymase type, as well as carboxypeptidase A3 [8–10].

In a previous, gene array-based study we found that several mast cell-related genes, in particular genes encoding chymases, were differently regulated in a hypervitaminosis A animal model for osteoporosis, introducing the possibility that mast cells may have a role in bone remodelling [11]. This notion is also supported by previous studies showing that mast cells accumulate close to bone surfaces in the marrow compartment during experimental hyperparathyroidism and rickets [12,13], after ovariectomy (OVX)-induced osteoporosis and following experimental fractures [14,15].

In fact, the term osteoimmunology was coined in 2000 to increase the awareness for the intimate connection between inflammatory diseases and accelerated bone loss [16]. Notably, although mast cells are best known for their involvement in allergy and anaphylaxis they also have important roles in the inflammatory process. Thus, mast cells produce inflammatory cytokines such as TNF and IL-6 which are known to stimulate bone resorption together with RANKL, the key transcription factor for osteoclastogenesis [17]. In addition, inflammatory mast cells have been linked to the pathology of fibrodysplasia ossificans progressive [18]. Along these lines, it is becoming increasingly clear that mast cell status in humans affects bone turnover [19] and since then a number of reports have pointed to a link between mastocytosis and reduced bone quality, particularly in men [20–23].

Importantly, although mast cells have been linked to bone remodelling by correlative observations, the functional impact of mast cells or of their products on bone phenotype has not been extensively evaluated. The aim of this study was to address this issue. Since our previous gene array-based study suggested that mast cell chymase was differently regulated in the hypervitaminosis A model [11], we considered chymase as being a likely candidate to have such a function.

In humans only one chymase gene is expressed (CMA1), whereas mice express a number of different chymase genes, out of which *Mcpt4* (also denoted mouse mast cell protease 4 (mMCP4)) represents the functional homologue to human chymase [7,24]. To address the possibility that mast cell chymase might have an impact on bone homeostasis we here evaluated the impact of Mcpt4-deficiency on bone phenotype. Our data reveal a marked increase in bone size and mass of *Mcpt4*^-/-^ vs. wild type (*wt*) mice, hence introducing mast cell chymase as a novel player in the regulation of bone remodelling.

## Results

### Female mice lacking Mcpt4 acquire wider femurs, while gaining less body weight, up to 12 month of age

**Fig 1:**
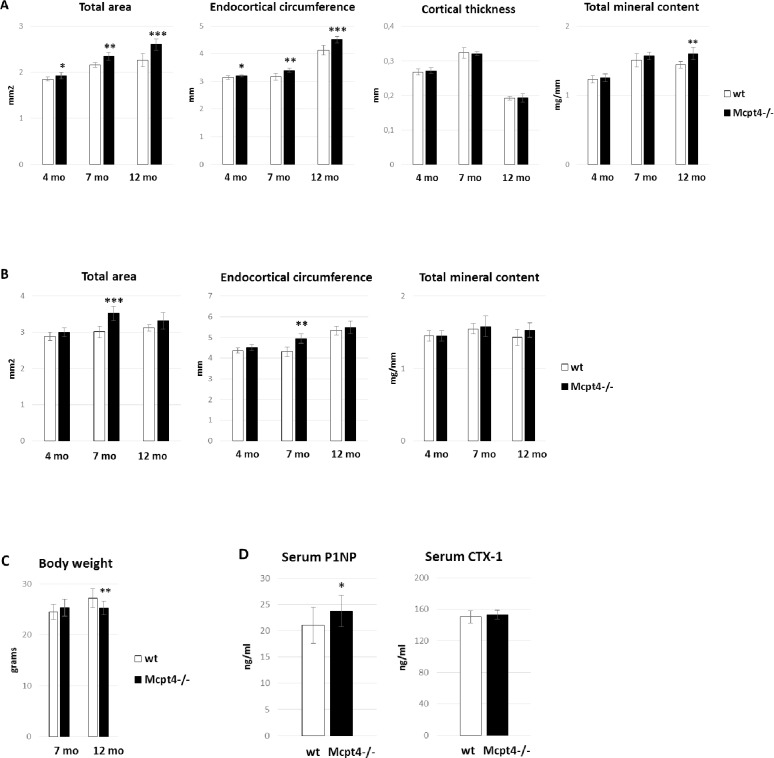
Bone phenotype and characteristics of *Mcpt4*^*-/-*^ female mice. **A)** Femur diaphyseal total cross sectional area (Total area), endocortical circumference, cortical thickness and total mineral content of *Mcpt4*^*-/-*^ females and *wt* controls, as determined by peripheral quantitative computed tomography (pQCT). The age and number of analysed animals were 4 months (wt, n = 9; Mcpt4-/-, n = 6), 7 (wt, n = 6; Mcpt4-/-, n = 5) and 12 (wt, n = 4; Mcpt4-/-, n = 9) months (mo) old. **B)** Femur metaphyseal parameters as described in (A). **C)** Body weight of *Mcpt4*^*-/-*^ females and *wt* controls at the age of 7 (wt, n = 12; Mcpt4-/-, n = 10) and 12 months (wt, n = 12; Mcpt4-/-, n = 20). **D)** Serum levels of P1NP (procollagen type 1 amino-terminal propeptide) and CTX-1 (C-terminal telopeptides of type I collagen), as determined by ELISA analysis (wt, n = 12; Mcpt4-/-, n = 12). Results are given as means ± SD. p < 0.05 *, p < 0.01 ** and p < 0.001 *** vs. *wt*.

As determined by using peripheral quantitative computed tomography (pQCT) analysis, female C57BL/6J mice lacking Mcpt4 (*Mcpt4*^*-/-*^) showed more pronounced progressive periosteal expansion than *wt* mice up to 12 months of age. Specifically, the diaphyseal bone cross sectional area (Total area) in *Mcpt4*^*-/-*^ mice was increased by 3.8 ± 0.036% (p = 0.029), 8.7 ± 0.037% (p = 0.0013) and 15 ± 0.053% (p = 0.00074) at the age of 4, 7 and 12 months, respectively, compared to *wt* controls (Fig 1A, S5 Fig, S1–S3 Tables). Although this was accompanied by a concomitant increase in endocortical circumference, the total mineral content was 11 ± 0.020% (p = 0.0061) higher in *Mcpt4*^*-/-*^ vs. *wt* mice at 12 months of age (Fig 1A). The cortical thickness was not affected by the absence of Mcpt4 (Fig 1A). At the metaphyseal site, periosteal expansion and increased endocortical circumference of *Mcpt4*^*-/-*^ mice was seen at the age of 7 months, although there was no significant effect on total mineral content (Fig 1B). By 12 months, *Mcpt4*^*-/-*^ mice developed a 7.0 ± 0.048% (p = 0.0021) lower body weight compared to *wt* controls (Fig 1C), suggesting that the increased bone volume of *Mcpt4*^*-/-*^ mice was not explained by a generally higher body mass (Fig 1C). Moreover, the lack of Mcpt4 did not result in altered femur length (S1 Fig). In agreement with an effect of Mcpt4 on bone formation, serum analysis of 12 month old mice indicated that the absence of Mcpt4 affects osteoblast activity, as evidenced by a slight increase in P1NP levels (Fig 1D). Notably, this was not accompanied with increased osteoclast activity as CTX-1 (C-terminal telopeptides of type I collagen) levels was unaffected in *Mcpt4*^*-/-*^ mice (Fig 1D). No effect of chymase-deficiency was seen on the serum levels of parathyroid hormone, calcium, phosphate or osteoclacin (S2 Fig).

### Female Mcpt4^-/-^ mice are not protected from bone loss induced either by ovariectomy (OVX) or excess vitamin A but was resistant from OVX-induced weight gain

**Fig 2:**
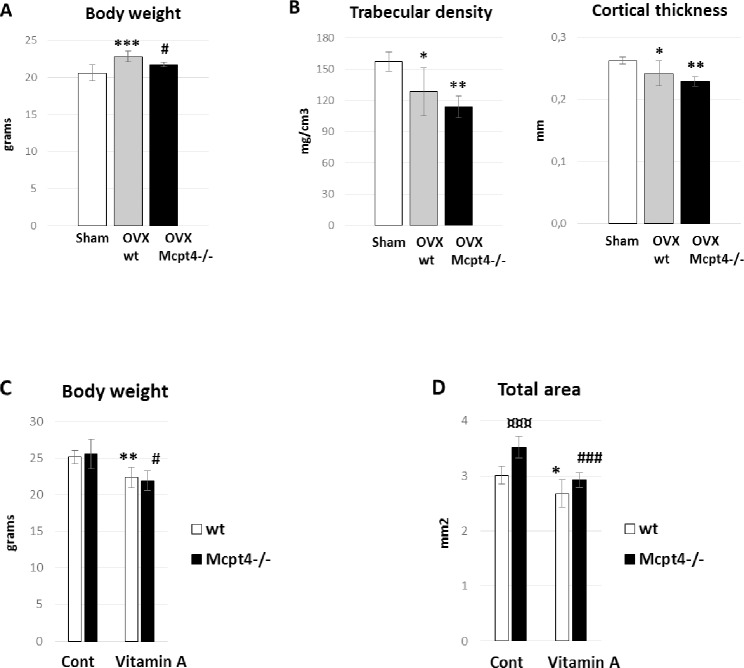
*Mcpt4*^*-/-*^ mice in ovariectomy- and vitamin A-induced osteoporosis models. **A)** Body weight 6 weeks after sham (n = 5) or ovariectomy (OVX) in *wt* (sham, OVX) (n = 7) and *Mcpt4*^*-/-*^ (OVX) (n = 4). **B)** Femur trabecular density and cortical thickness in animals as described in (A). **C)** Body weight after 1 week of hypervitaminosis A at the age of 7 months. Number of animals in each group are wt controls (Cont, wt; n = 6), Mcpt4-/- controls (Cont, Mcpt4-/-; n = 5), controls vitamin A (Vitamin A, wt; n = 5) and vitamin A Mcpt4-/- (Vitamin A, Mcpt4-/-; n = 5). **D)** Femur diaphyseal total cross sectional area (Total area) after 1 week of hypervitaminosis A. Results are given as means ± SD. p < 0.05 *, p < 0.01 ** and p < 0.001 *** vs. *wt/sham/Cont*., p < 0.05 **#** and p < 0.001 **###** vs. Cont *Mcpt4*^*-/-*^, p < 0.001 **¤¤¤** vs. Cont *wt*.

To further evaluate the effect of chymase on bone phenotype, we investigated whether mice lacking Mcpt4 were protected from OVX-induced bone loss. OVX mimics postmenopausal bone loss and primarily affects trabecular bone and endocortical bone surfaces. Six weeks after OVX, *wt* mice showed the expected body weight gain (11 ± 1.2%, p = 0.00042). In contrast, mice lacking Mcpt4 were resistant towards this weight gain (Fig 2A). Analysis using pQCT of bone changes induced by OVX showed loss of trabecular density (18 ± 6.7%, p = 0.012) and cortical thickness (8 ± 3.1%, p = 0.029) in *wt* controls compared to sham-operated *wt* mice (Fig 2B). Mice lacking Mcpt4 were not protected from OVX induced bone loss and showed reduced trabecular density (26 ± 4.7%, p = 0.0022) and cortical thickness (13 ± 1.7%, p = 0.0038) compared to sham-operated *wt* mice (Fig 2B).

Next, we used hypervitaminosis A as an alternative osteoporosis model as it is different from OVX-induced osteoporosis by primarily affecting the periosteal bone surface [25]. After one week of excess vitamin A ingestion, *wt* mice showed the classical signs of hypervitaminosis A with reduced weight gain (11 ± 2.8%, p = 0.0032) and reduced total area (11 ± 4.1%, p = 0.024)(Fig 2C and 2D). Mice lacking Mcpt4 were not protected from either vitamin A-induced weight loss (14 ± 2.7%, p = 0.011) or bone thinning, i.e. reduced total area (17 ± 2.0%, p = 0.00048)(Fig 2C and 2D). However, in agreement with the data displayed in Fig 1A, the baseline total area was markedly higher in *Mcpt4*^*-/-*^ vs. *wt* mice (Fig 2D).

### Female mice lacking Mcpt4 show opposite signs of osteoblast activity at the periosteal and endosteal surfaces together with altered bone microarchitecture

**Fig 3:**
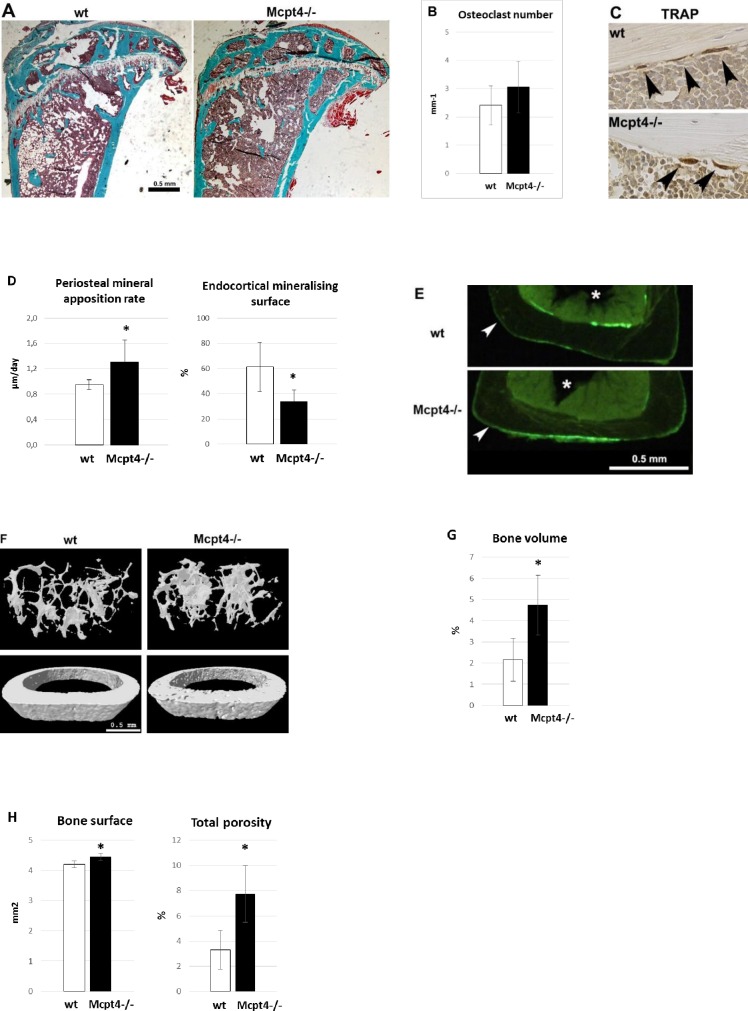
Histomorphometric and μCT analysis of bone tissue. **A)** Representative images of Masson-Goldner Trichrome stained undecalcified tibia sections used for static histomorphometric analysis. **B)** Static histomorphometric analysis of osteoclast numbers at tibia metaphysis of *Mcpt4*^*-/-*^ (n = 5) and *wt* (n = 5) mice. **C)** Immunostaining for tartrate resistant phosphatase (TRAP) in endocortical osteoclasts at the diaphysis of humerus. **D)** Dynamic histomorphometric analysis from calcein double labelling showing diaphyseal periosteal mineral apposition rate and endocortical mineralising surface in tibias from *Mcpt4*^*-/-*^ (n = 5) and *wt* (n = 5) mice. **E)** Representative low power images of calcein labelling areas in undecalcified tibial bone sections of wt and Mcpt4-/- mice. Arrowhead highlight periosteal surfaces and asterisks mark the marrow compartments (bar = 0.5 mm). **F)** Representative images from μCT (micro computed tomography) analysis of femur. **G)** Bone volume of femur metaphysis as determined by high resolution μCT measurements of *Mcpt4*^*-/-*^ (n = 3) and *wt* (n = 4) mice. **H)** Bone surface and cortical total porosity as determined by μCT measurements at femur diaphysis of *Mcpt4*^*-/-*^ (n = 3) and of *wt* (n = 4) mice. Results are given as means ± SD. p < 0.05 * vs. *wt* controls.

To further substantiate the effect of chymase on bone parameters we next implemented histomorphometric analysis of Masson-Goldner Trichrome stained undecalcified tibia bone sections (Fig 3A). Static histomorphometric analysis did not reveal any statistical significant differences in osteoclast or osteoblast numbers at the metaphysis (Fig 3B and S3 Fig). In addition, immunostaining of endocortical osteoclasts for TRAP (tartrate resistant acid phosphatase) at the diaphysis did not reveal any obvious differences in size or staining intensity between osteoclasts in *Mcpt4*^*-/-*^ compared to *wt* bone (Fig 3C). Further, analysis based on *in vivo* calcein double labelling experiments revealed an increased periosteal mineral apposition rate (38 ± 12%, p = 0.032) in *Mcpt4*^*-/-*^ vs. *wt* mice (Fig 3D, left panel), while the endocortical mineralising surface (55 ± 12%, p = 0.018) was reduced (Fig 3D, right panel). In addition, representative overview pictures of calcein labelled transverse sections show more periosteal label in Mcpt4^-/-^ bone in contrast to *wt* bone which has more endosteal label (Fig 3E). Furthermore, and in line with the pQCT results, high resolution μCT analysis (Fig 3F, S4 Table) revealed increased bone volume in *Mcpt4*^*-/-*^ vs. *wt* mice, with a femur metaphyseal bone volume of 4.7 ± 0.66% compared to 2.2 ± 0.47% in *wt* controls (p = 0.0363)(Fig 3G, S5 Fig). At the femur diaphysis, μCT analysis showed that *Mcpt4*^*-/-*^ bones have an increased bone surface but also increased cortical total porosity (7.7 ± 2.2% vs. controls: 3.3 ± 1.5%, p = 0.017)(Fig 3H).

### Increased mast cell degranulation around *Mcpt4*^*-/-*^ bone

**Fig 4:**
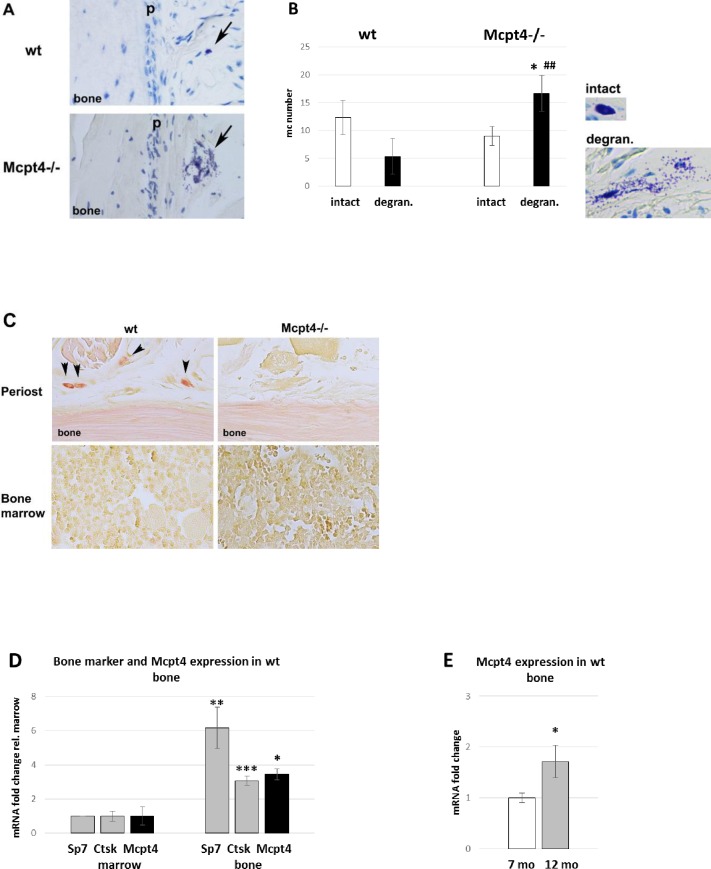
Mast cell and Mcpt4 expression in long bones. **A)** Arrows highlight mast cells close to periosteum (p) in Toluidine blue-stained sections of humerus of 12 month-old animals. **B)** Counting and determination of degranulation of Toluidine blue stained mast cells around of *Mcpt4*^*-/-*^ (n = 3) and of *wt* (n = 4) bone as described in (A). **C)** Chloroacetate esterase-staining (red) stains Mcpt4 activity in *wt* mast cells (arrowheads, n = 3) but not in *Mcpt4-/-* mast cells (right panel, n = 4), on sections as described in (A). **D)** qPCR analysis of transcripts of the osteoblast marker Sp7 (osterix), the osteoclast marker Ctsk (cathepsin K) and of Mcpt4 (mast cell protease 4) in preparations of bone (n = 3) and marrow tissue (n = 3) of *wt* controls. **E)** qPCR analysis of the transcript levels of Mcpt4 in *wt* control at 7 months (n = 3) and 12 months (n = 4). Results are given as means ± SD. p < 0.05 * vs. intact *Mcpt4*^*-/-*^, p < 0.01 **##** vs. degran. *wt*. (4B); p < 0.05 *, p < 0.01 ** and p < 0.001 *** vs. marrow (4D); p < 0.05 * vs 7 mo (4E).

Histological examination using Toluidine blue (stains mast cell granules), demonstrated that mast cells were scattered around the periosteal surface of control mouse bones, whereas the bone marrow was devoid of mature mast cells (Fig 4A). A quantification of mast cell numbers in decalcified bone sections did not reveal any statistically significant difference in the total numbers of mast cells when comparing bones from *wt* and *Mcpt4*^*-/-*^ mice. However, the number of degranulated mast cells was increased around *Mcpt4*^*-/-*^ bones (Fig 4B), with approximately two thirds of mast cells showing signs of degranulation, whereas in controls only one third of the mast cells close to bone appeared degranulated. Hence, these findings suggest that the absence of chymase is associated with a higher state of mast cell activation.

To specifically depict chymase activity in bone, we used chloroacetate esterase-staining on decalcified paraffin-imbedded bone sections. In control tissue, clear staining was seen in mast cells close to the periosteal bone surface, whereas bone marrow cells were devoid of distinct staining (Fig 4C). Importantly, no chloroacetate esterase-positive cells could be found in bone tissue from *Mcpt4*^*-/-*^ mice (Fig 4C), verifying that the chloroacetate esterase-staining procedure specifically stains for chymase. Importantly, these findings also demonstrate that Mcpt4 represents the major enzyme with chymase-like activity in bone tissue.

### Mcpt4-deficiency causes altered expression of genes affecting bone remodelling

A further indication that mast cells are intimately associated with bone cells was the observation that Mcpt4 transcripts associate with both osteoblast and osteoclast transcripts upon isolation of bone tissue. Thus, *Mcpt4* expression follows closely the expression of the osteoblast transcription factor Sp7 (osterix) and the osteoclast protease Ctsk (cathepsin K) during separation of bone tissue from bone marrow, a tissue known to be devoid of mast cells in mice (Fig 4D). Furthermore, Mcpt4 expression is higher in older bone as determined by qPCR (Fig 4E). Notably, *Mcpt4* is a highly mast cell-specific gene, as we noticed a 340-fold higher expression in mast cell preparations compared to expression in mouse osteoblastic cells (MC3T3-E1) and we were unable to detect *Mctp4* expression in mouse osteoclast precursors or mature osteoclasts *in vitro* (RAW 264.7)(S4 Fig).

**Fig 5:**
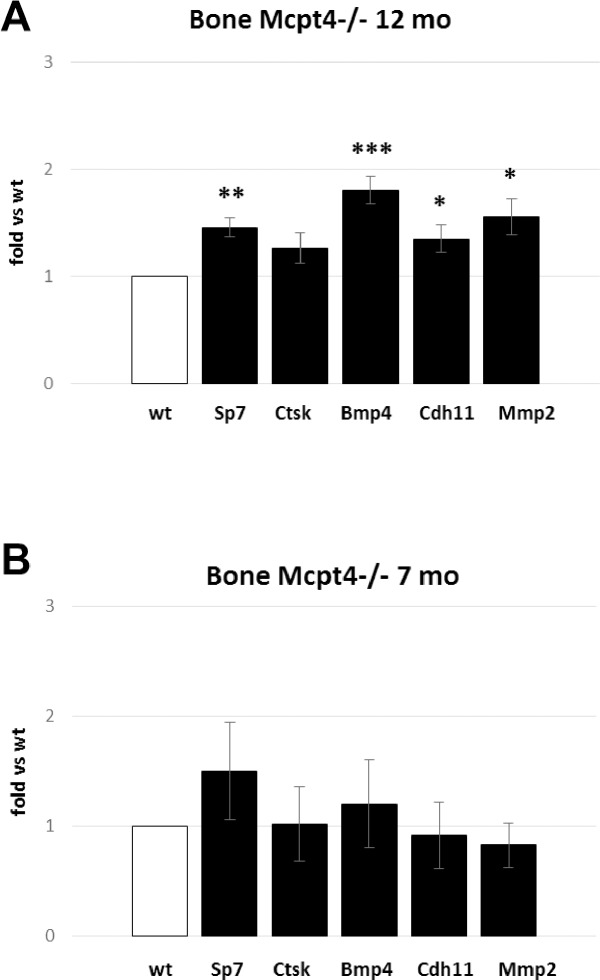
Transcript analysis of bone associated genes. **A)** Differences, at the age of 12 months, in expression of genes associated with bone growth in *Mcpt4*^*-/-*^ bone tissue (n = 4) compared to *wt* mice (n = 4); osteoblast marker Sp7 (osterix), the osteoclast marker Ctsk (cathepsin K), Bmp4 (bone morphogenetic protein 4), Cdh11 (cadherin 11) and Mmp2 (matrix metalloprotease 2). **B)** Differences, at the age of 7 months, in expression of genes associated with bone growth in *Mcpt4*^*-/-*^ bone tissue (n = 3) compared to *wt* mice (n = 3) as described in (A). Results are given as means ± SD. p < 0.05 *, p < 0.01 ** and p < 0.001 *** vs. *wt*.

To provide additional insight into the mechanism by which the absence of chymase leads to increased bone mass, we evaluated whether chymase-deficiency affected the expression of a few selected genes linked to bone remodelling and size. As seen in Fig 5A, qPCR analysis revealed a slight but significant elevation of the osteoblast marker Sp7 in *Mcpt4*^*-/-*^ bones, suggesting that the lack of chymase leads to increased osteoblast activity. The absence of chymase was also accompanied by an upregulated expression of bone morphogenetic protein 4 (Bmp4), which is linked to hip bone density in postmenopausal women [26], improved experimental fracture healing [27] and with mast cell-associated excessive pathological bone formation in fibrodysplasia ossificans progressive [18]. Upregulation was also seen for cadherin 11 (Cdh11), a strong predictor of bone size [28] and for matrix metalloprotease 2 (Mmp2); the latter enzyme is expressed mainly in osteoblasts/osteocytes in bone and has positive effects on bone formation [29] and osteocyte canalicular formation [30]. The expression of Ctsk, an osteoclast marker, was not significantly different from *wt* controls. Together, these data indicate that the absence of chymase leads to multiple positive effects on genes involved in bone growth. Importantly, the upregulation of these genes was linked to the aging of the mice, as neither of the assessed genes were significantly upregulated in younger animals (Fig 5B).

## Discussion

Mast cells are well-established effector cells of the immune system, contributing to both innate and adaptive immune responses. Consequently, the general notion is that mast cells predominantly have an impact on its host during conditions when the immune system has been triggered, for example in various pathological settings [4–6,31]. In line with this notion, mast cells and their secreted proteases have been shown to play an important role in various pathological settings involving the immune system, and in many cases it has been demonstrated that mast cell chymase accounts to a large extent for the impact of mast cells under such circumstances [7]. For example, chymase has a prominent role in allergic airway inflammation [32], arthritis [33], abdominal aortic aneurysm formation [34], skin blistering [35], atherosclerosis [36], brain inflammation [37] and glomerulonephritis [38].

In contrast to the established role of mast cells and their products during immune responses, the possibility that they can have an impact on normal tissue homeostasis has not been extensively investigated. Here we provide evidence that one of the mast cell products, chymase, regulates bone mass in the absence of immunological triggers. Clearly, these findings expand the repertoire of mast cell functions to include important regulatory effects on normal tissue homeostasis. Notably, a homeostatic impact of chymase is also supported by previous studies showing that chymase has an impact on normal connective tissue homeostasis of the skin [39] and on baseline intestinal barrier function [40]. Interestingly, the effect of chymase on bone remodelling appears to be restricted to normal bone homeostasis, as no significant protection due to chymase-deficiency was seen in either of two different models of experimental osteoporosis (OVX and hypervitaminosis A).

Our data show that female chymase-deficient mice have increased periosteal bone expansion concomitant with reduced body weight. By the age of 12 months, the diaphyseal cross sectional area was 15% larger in *Mcpt4*^*-/-*^ mice than in *wt* controls while their body weight was 7% less. This diaphyseal bone expansion resulted in an 11% higher total mineral content. In line with these data, we noticed an increased mineral apposition rate at the periosteal bone surface and increased serum levels of the anabolic bone marker P1NP. Furthermore, in agreement with the strong age-dependency for the chymase effect, it has been shown that the numbers of bone mast cells increase profoundly as mice undergo aging [41].

Several of our findings suggest that the absence of chymase leads to increased osteoblast activity. This was first indicated through serum marker analysis. In addition, gene expression analysis of bone tissue revealed that the expression of the osteoblast marker Sp7 and the bone size-linked gene, Cdh11 were elevated in *Mcpt4*^*-/-*^ vs. *wt* animals. These findings together with the *in vivo* calcein double labelling experiments are in agreement with increased bone building activity, resulting in a persistent increase in bone size and mass in *Mcpt4*^*-/-*^ mice vs. *wt* controls. Altogether, these data suggest that the absence of chymase may result in a general stimulatory effect on osteoblast activities, leading to an increase in bone mass.

Intriguingly, it has been recognized for a long time that mastocytosis, i.e. conditions with expanded mast cell populations, is linked to osteoporosis [19,42]. It is notable that, differently to osteoporosis in general, there is a higher prevalence of osteoporosis in men compared to women in patients with indolent systemic mastocytosis [20]. Importantly, though the connection between mastocytosis and osteoporosis is well documented, the underlying mechanism behind this association has not been revealed. Based on the findings presented here, we may propose that mast cell chymase might contribute to the detrimental impact of mastocytosis on bone.

The mechanism by which chymase regulates bone mass is intriguing. One likely scenario would be that mast cells release growth factors that promote bone building, e.g. by enhancing osteoblast activity. In line with such an activity of mast cells, we noted that mast cells are strategically located around the periosteal surface. Thereby, mast cells are in close anatomical vicinity to periosteal osteoblasts, the cells responsible for the bone expansion. In further agreement with such a scenario, mast cells are known to express a wide panel of growth factors, including factors that potentially could influence cells involved in bone remodelling. In fact, there appears to be a link between mast cell- and osteoblast-activity as ligation of bone morphogenetic protein receptor, type 1A (BMPR1A, the preferred receptor for Bmp4) on mast cells enhances their activation capacity and survival [43]. In addition, bone-synthesizing osteoblasts produce stem cell factor, a stimulator of mast cell development [44], suggesting the presence of a positive feedback mechanism between bone formation and mast cell activation. Along this line we also noticed increased expression of two additional positive factors for bone strength/size—Cdh11 [28,45] and Mmp2 [29] in bones from *Mcpt4*^*-/-*^ mice, whereas the expression of the osteoclast marker Ctsk was not altered. Possibly, there is a link between increased Mmp2 expression, an important gene for bone canaliculi formation, and the increase in cortical porosity. In particular, Cdh11 is recognized as a strong candidate gene for regulating femoral morphology as lack of its expression in mice results in thinner bones. In line with this we found that bones from mice devoid of chymase activity have wider bones and higher expression of Cdh11. Additionally, we observed in this study that the absence of chymase was accompanied by a much higher tendency of the bone-proximal mast cells to degranulate, concomitantly with a higher expression of *Bmp4* in bone. The Bmp family of proteins are pleiotropic but are mostly known for their potent induction of bone formation. More specifically, osteoblastic *Bmp4* expression is necessary for normal osteoblast function [46] and its overexpression has been shown to improve healing of experimental fractures [27]. Although we cannot with certainty explain the mechanism by which chymase regulates mast cell degranulation in bone, one possibility would be that chymase can degrade mast cell-activating compounds, this being in line with the demonstrated role of chymase in degrading various cytokines and alarmins [32,47–50]. The increased bone mass in chymase-deficient animals could thus be explained by increased mast cell degranulation, resulting in enhanced release of factors affecting bone remodelling.

Based on the findings presented here, it is possible that chymase inhibition could represent a possible novel strategy for therapeutic intervention to enhance bone mass. Several chymase inhibitors have to date been developed and have been frequently used in animal models of disease [51]. In many cases, a beneficial effect of such compounds has been noted, for example in atherosclerotic plaque progression [52], myocardial infarction [53], atopic dermatitis [54], pulmonary inflammation [55], peritoneal adhesion formation [56] and abdominal aortic aneurysm formation [57]. However, to possess efficacy in regulating bone mass, it is essential that such inhibitors show high bioavailability, penetrating into the bone. However, considering that most chymase inhibitors developed to date are poorly soluble in aqueous solutions, this may represent a substantial challenge.

## Materials and Methods

### Mice and experimental design

This study was carried out in strict accordance with the recommendations in the Guide for the Care and Use of Laboratory Animals of the National Institutes of Health. The protocol was approved by the Committee on the Ethics of Animal Experiments of the University of Uppsala, Sweden (Permit Number: C275/12) and specifically approved this study. Animals were housed in a conventional animal facility. All surgery was performed under sodium pentobarbital anesthesia, and all efforts were made to minimize suffering. Mice deficient in *Mcpt4* expression [24] were backcrossed for 13 generations to the C57BL/6J genetic background. Age-matched *wt* animals were used as controls. Female mice were used for all experiments. Animals were bred and maintained in the animal facility at The Swedish Veterinary Agency (SVA). Mice were sham operated (SHAM) or ovariectomized (OVX) at the age of 9 weeks and killed 6 weeks after surgery. Temgesic (0.05–0.1 mg/kg) was administered 30 minutes before start of OVX surgery and each 8-12h for 48h after surgery. To induce hypervitaminosis A, 30 week-old mice were fed a diet supplemented with 1700 IU vitamin A/g pellet (standard diet containing 12 IU vitamin A/g pellet) for one week. The vitamin A was added to the pellets in the form of retinyl palmitate and retinyl acetate as described previously [25]. Body weight was measured at the beginning of each experiment (initial weight) and before the mice were killed (final weight). Blood was collected from one eye during anesthesia followed by euthanasia via cervical dislocation. Blood (approximately 0.3 mL) was left at room temperature for 30 min before centrifuging at 200×g for 10 min to separate sera, individual aliquots were stored at −70°C pending biochemical analyses.

### Peripheral quantitative computed tomography (pQCT)

The left femora were scanned using a Stratec XCT Research SA+ apparatus with version 5.50 R software (Norland Stratec Medizintechnik GmbH, Birkenfeld, Germany) using a voxel size of 0.07 mm and a scan speed of 3 mm/s. The sites of the scans were 20% from the condyle for metaphysis and 50% of length for diaphysis (S5 Fig). All scans were performed post mortem.

### Micro-computed tomography (micro-CT)

This analysis was conducted by Pharmatest Services Ltd, Turku Finland. Micro-CT parameters were measured from distal metaphysis (trabecular bone) and diaphysis (cortical bone) of femur with 5.3 micrometer resolution using Skyscan 1172 equipment with analysis software CTan (Skyscan, Belgium). Region of interest of trabecular bone was selected below the growth plate approximately 2 mm from the distal end of femur and contained 200 slices (1.06 mm). Region of interest of cortical bone was selected approximately 6.2 mm from the distal end of femur (S5 Fig).

### Determination of serum markers

Commercially available kits were utilized for measurement of serum levels as follows: osteocalcin, Rat-MID TM Osteocalcin (Nordic Bioscience Diagnostics, Herlev, Denmark); C-terminal telopeptides of type I collagen (CTX-1), RatLaps TM (Nordic Bioscience Diagnostics); N-terminal propeptide of type I collagen, PINP EIA (Immunodiagnostic Systems, Boldon, UK); intact PTH ELISA (Immutopics Inc., San Clemente, CA, USA); phosphate, QuantiChromeTM phosphate assay kit (BioAssays Systems, CA, USA) and Ca^2+^, Calcium colometric assay kit (BioVision Milpitas USA).

### Histomorphometry, histology and immunohistochemistry

The bones from all animals were sectioned in the same orientation in order to make comparable sections. Histomorphometry analysis was conducted on the tibial bone by Pharmatest Services Ltd, Turku Finland. The static histomorphometric analysis of trabecular bone was performed using OsteoMeasure software version 2.2 (Osteometrics, Atlanta, GA, USA) and the histomorphometric analysis of cortical bone using BioQuant Osteo II software version 8.12 (BioQuant Image Analysis Corporation, Nashville, TN, USA) on Masson-Goldner Trichrome stained undecalcified bone sections. For the measurement of dynamic parameters, bones were labelled with calcein at day 0 and day 6 prior to the scheduled terminal necropsy at day 8. For histology and immunohistochemistry the humeral bones were used; fixed in 4% paraformaldehyde, demineralized, paraffin embedded, and cut into 6-μm sections. One representative decalcified bone section from 3–4 different animals per group was analysed. Numbers and morphology of mast cells were evaluated by examining toluidine blue stained sections. All cells between the growth plate and 2.5 mm below were counted. Chymotryptic activity was detected by chloroacetate esterase-staining on decalcified sections as described earlier [58]. Immunostaining for tartrate resistant acid phosphatase (TRAP) was achieved by the use of polyclonal anti-mouse TRAP antiserum at a dilution of 1:300 [59]. Visualization of the antibodies was achieved by incubation with secondary biotinylated antibody at a dilution of 1:200 in PBS/10% serum, followed by incubation with avidin–peroxidase and detection of peroxidase activity using the Vectastain ABC-kit (Vector Laboratories) and the substrate diaminobenzidine tetrahydrochloride (DAB, DAKO).

### Bone tissue RNA Isolation

Humeri were isolated, and all connective tissue, including periosteum, was completely removed, as were both epiphyses, including growth plates, as previously described [25]. Marrow was flushed out with ice cold PBS, collected by centrifugation followed by immersion in TRI Reagent (Sigma-Aldrich, St. Louis, MO, USA) for total RNA extraction. The remaining bone sample was snap frozen in liquid nitrogen and quickly crushed into a fine powder using a mortar and pestle, followed by total RNA extraction with TRI Reagent. Isolated RNA was quantified using spectrophotometry by measuring the absorbance at 260 nm, and the 260/280 nm ratio was calculated. This ratio was 1.9–2.0 in all samples, indicating the absence of protein contamination. The integrity of sample RNAs was confirmed by capillary electrophoresis separating 18 S and 28 S ribosomal RNA on an Agilent Technologies (Palo Alto, CA) 2100 Bioanalyzer. Notably, the metaphyses and growth plates were not included in these samples.

### Quantitative RT-PCR

Four hundred ng of total RNA was transcribed to cDNA using the TaqMan system (Applied Biosystems, USA). Quantitative real time PCR was performed using inventoried TaqMan Gene Expression Assays for; Sp7 (Osterix) ENSMUSG00000060284 (Mm00504574_m1); Ctsk (cathepsin K) ENSMUSG00000028111 (Mm00484036_m1); Bmp4 (bone morphogenetic protein 4) ENSMUSG00000021835 (m00432087_m1); Cdh11 (cadherin 11) ENSMUSG00000031673 (Mm00515466_m1) and Mmp2 (matrix metalloprotease 2) ENSMUSG00000031740 (Mm00439498_m1) according to the manufacturer’s protocol, on a CFX96 Touch™ Real-Time PCR Detection System apparatus. Cycling protocol: 50°C for 2 min, followed by 95°C for 10 min and then 40 cycles of 95°C 15 sec followed by 60°C for 1 min. For standardization, expression levels were divided by expression level for Actb ENSMUSG00000029580 (Mm00607939_s1), derived from dilution standard curves of Ct values for each gene. Each experiment was performed at least three times using triplicates.

### Statistical analyses

The data were analysed by the Students t-test or, for variables with deviations from the normal distribution, the Mann-Whitney U test. In every case, p < 0.05 was considered statistically significant. ANOVA with the Post-hoc test Fisher LSD was used for the OVX analysis.

## Supporting Information

S1 Fig Femur length. (TIF)

S2 Fig Additional serum analysis. (TIF)

S3 Fig Osteoblast number. (TIF)

S4 Fig Mcpt4 mRNA analysis in osteoclastic cells. (TIF)

S5 Fig Region of interest (ROI) in bone CT analysis. (TIF)

S1 Table Femur pQCT characteristics of 4 mo old female mice. (DOCX)

S2 Table Femur pQCT characteristics of 7 mo old female mice. (DOCX)

S3 Table Femur pQCT characteristics of 12 mo old female mice. (DOCX)

S4 Table Femur micro CT characteristics of 7 mo old female mice. (DOCX)
