# Supplementary figures and images for: Increased Bone Mass in Female Mice Lacking Mast Cell Chymase
Source: PLoS One. 2016 Dec 9;11(12):e0167964. doi: 10.1371/journal.pone.0167964 (PMC5148084; doi:10.1371/journal.pone.0167964)

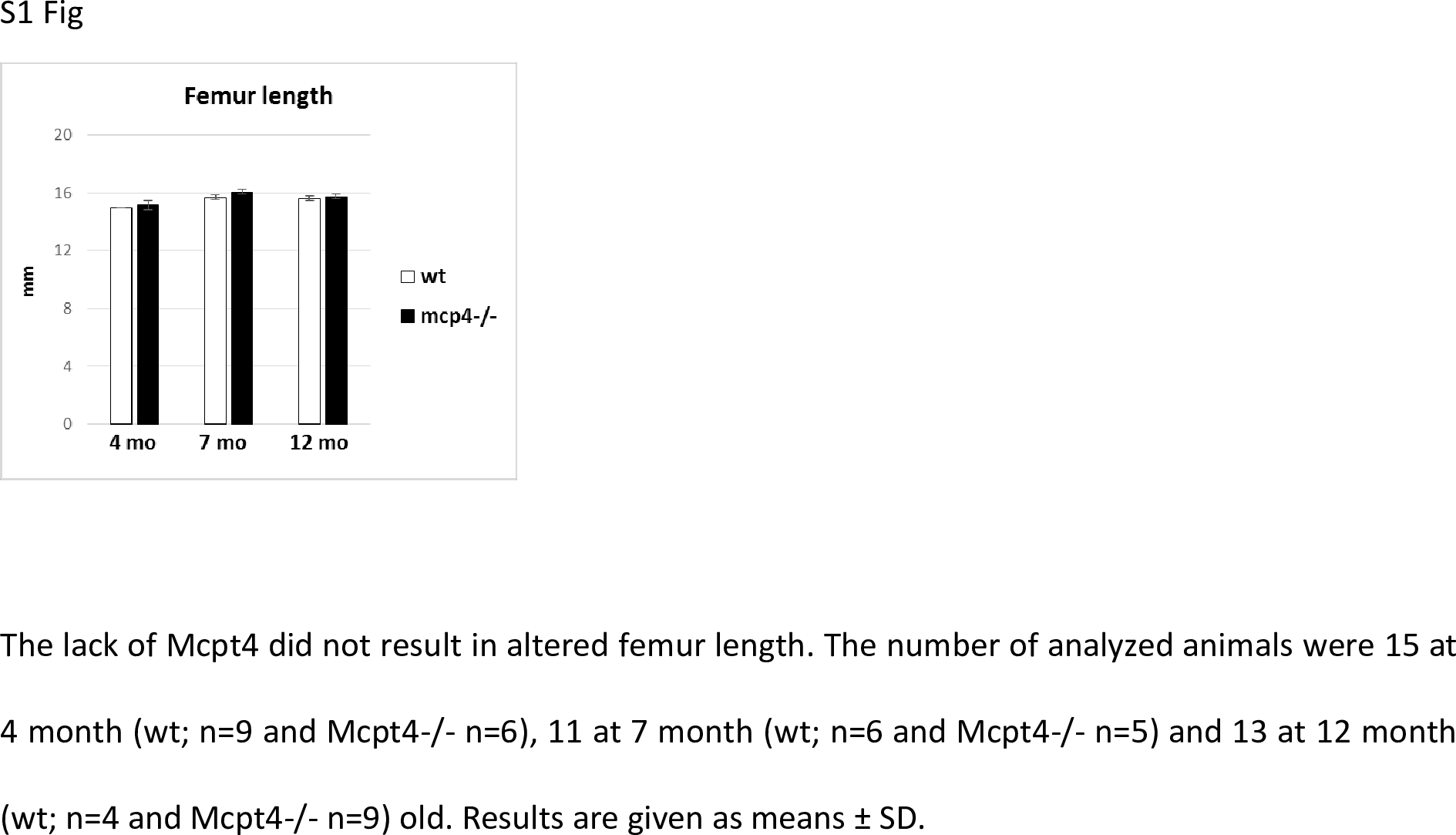

Supplement: S1 Fig — (TIF) [file pone.0167964.s001.tif]

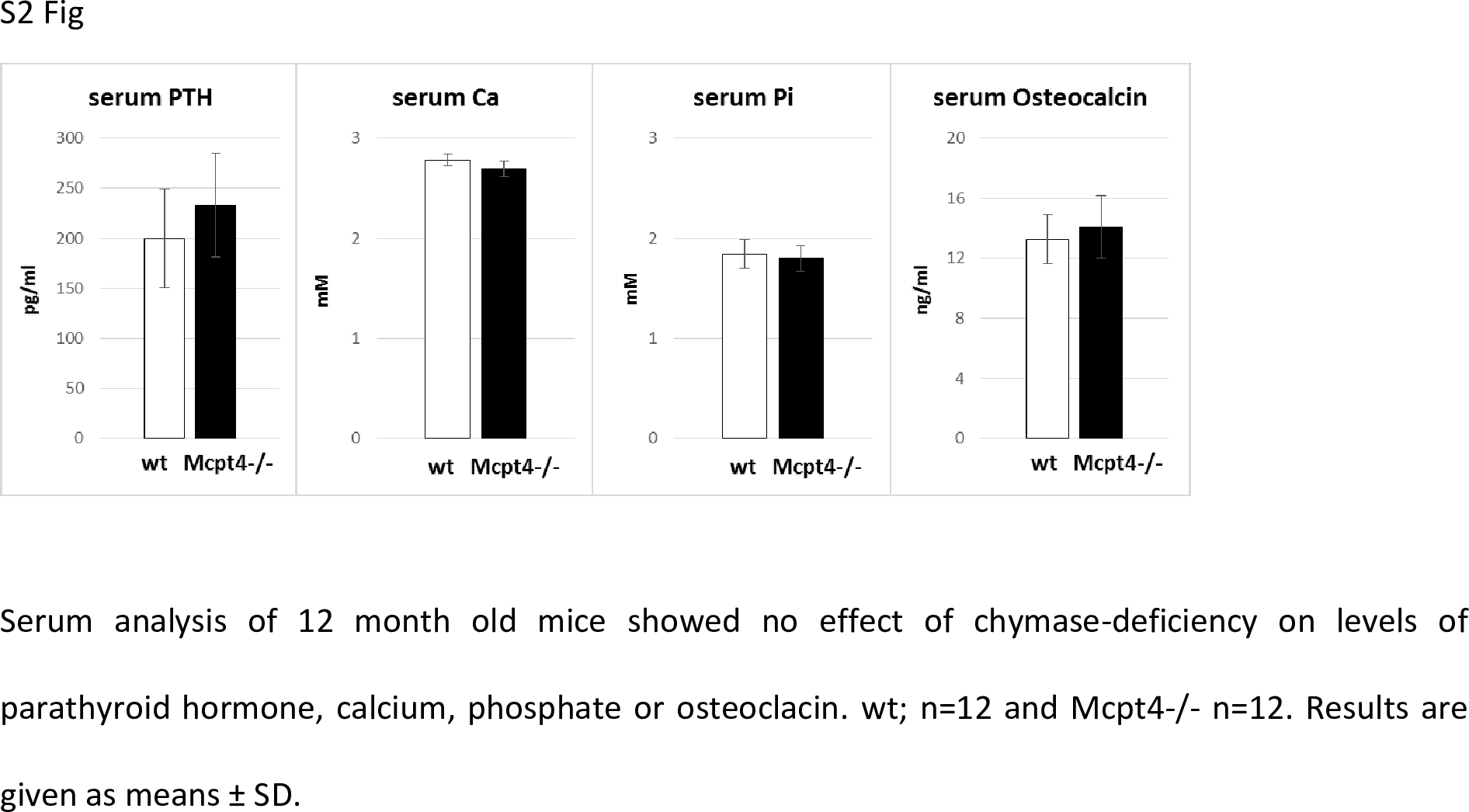

Supplement: S2 Fig — (TIF) [file pone.0167964.s002.tif]

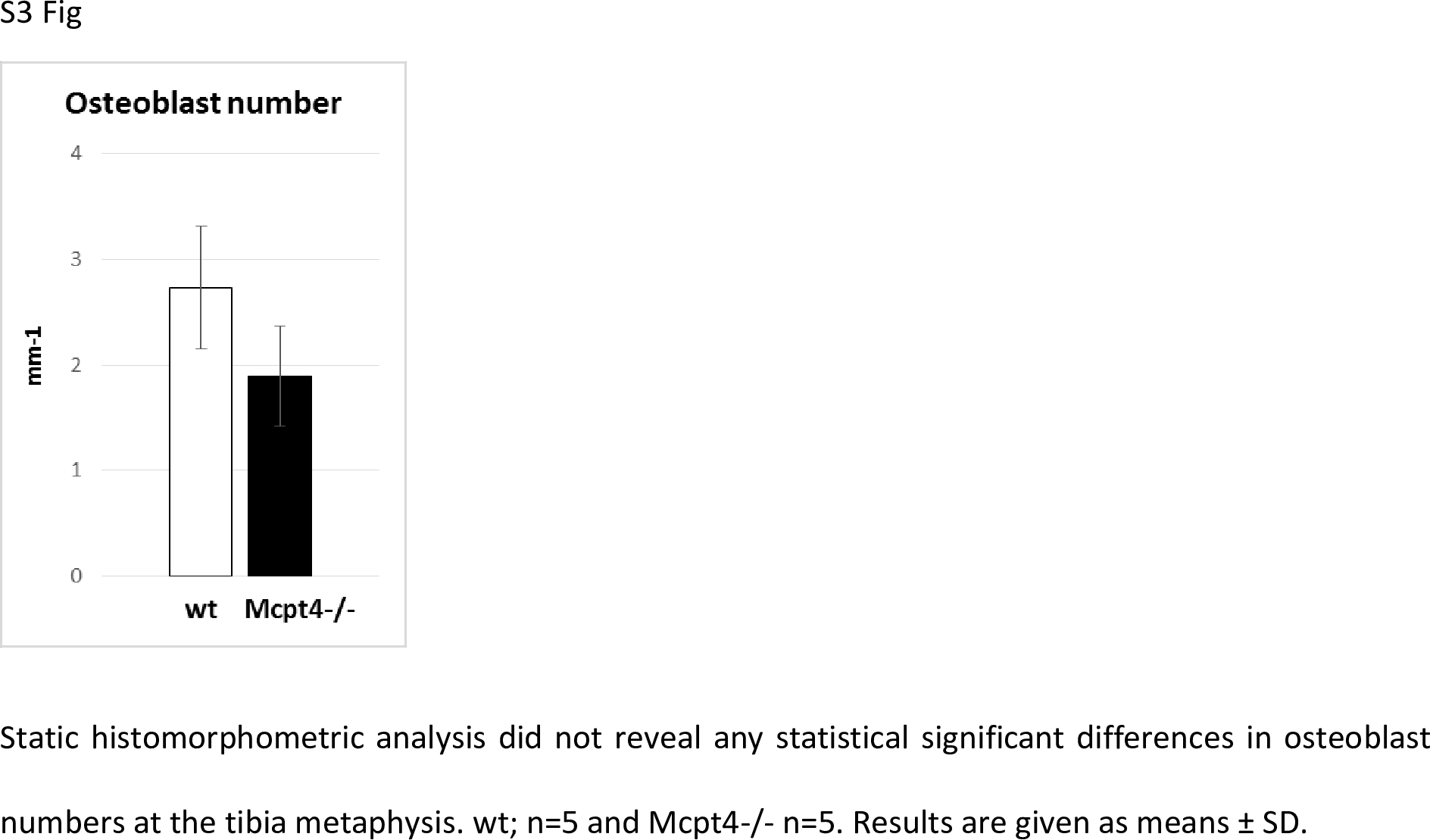

Supplement: S3 Fig — (TIF) [file pone.0167964.s003.tif]

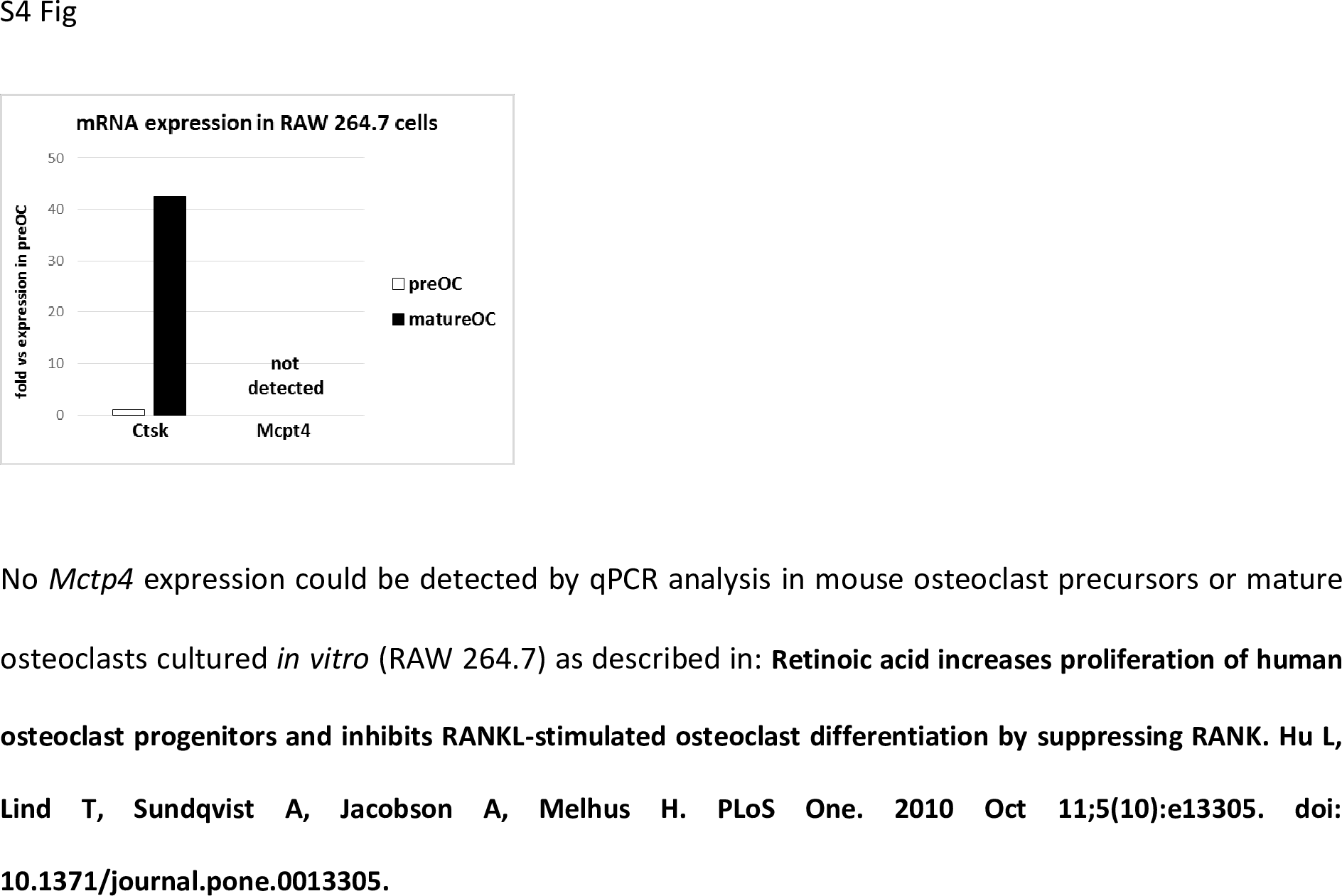

Supplement: S4 Fig — (TIF) [file pone.0167964.s004.tif]

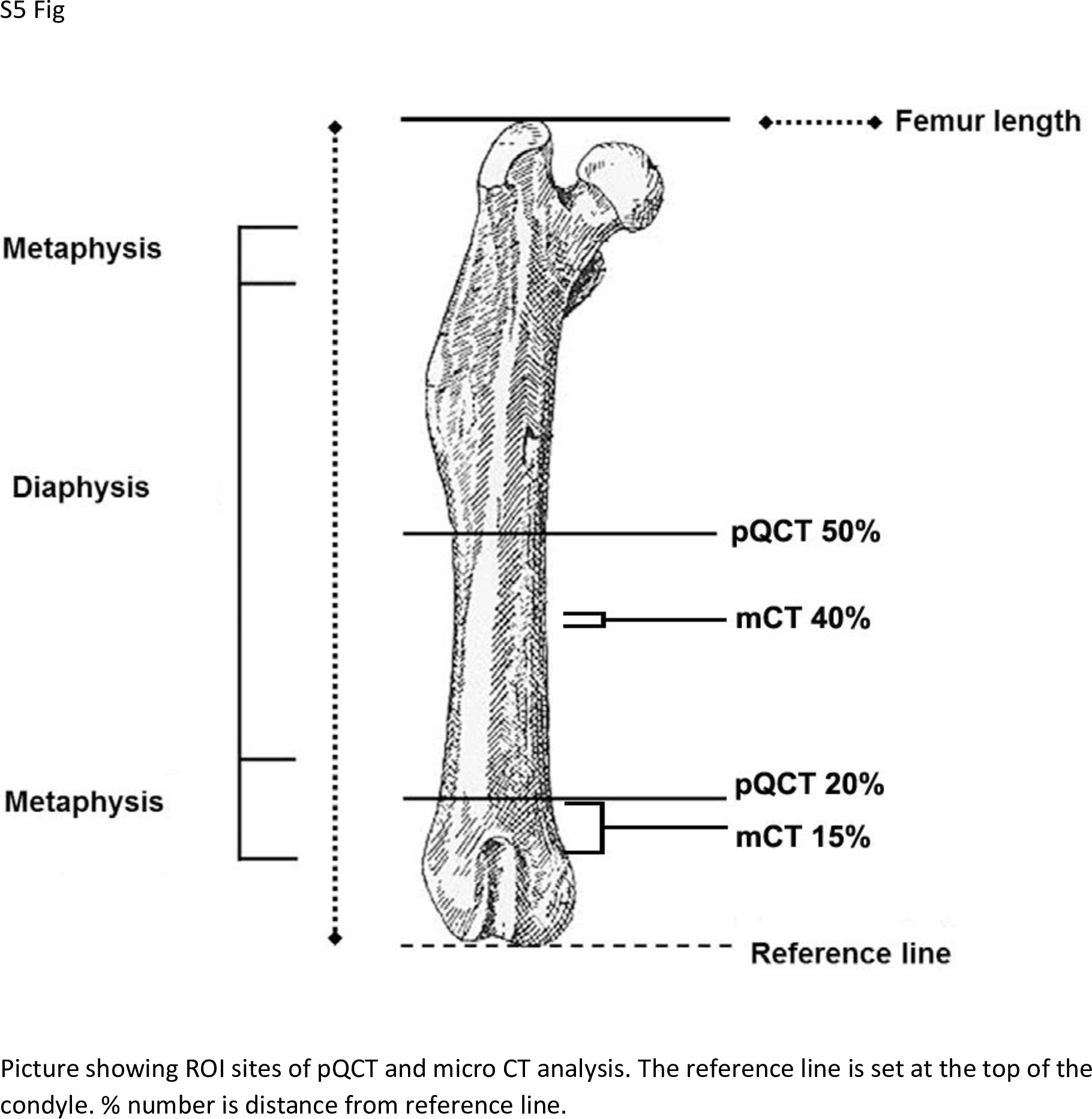

Supplement: S5 Fig — (TIF) [file pone.0167964.s005.tif]
