# Supplementary material for: Increased Bone Mass in Female Mice Lacking Mast Cell Chymase
Source: PLoS One. 2016 Dec 9;11(12):e0167964. doi: 10.1371/journal.pone.0167964 (PMC5148084; doi:10.1371/journal.pone.0167964)
Supplement: S1 Table — (DOCX) [file pone.0167964.s006.docx]

S1 Table. Femur pQCT characteristics of female mice.

|  | **WT 4 mo**  n=9 | **Mcpt4-/- 4 mo**  n=6 | **p-value** |
| --- | --- | --- | --- |
| Femur length (mm) | 15.0 ± 0.088 | 15.2 ± 0.73 | 0.4325 |
| ***Distal metaphysis*** |  |  |  |
| TOT_CNT (mg/mm)  TOT_DEN (mg/cm³)  TRAB_CNT (mg/mm)  TRAB_DEN (mg/cm³)  TOT_A (mm²)  TRAB_A (mm²)  ENDO_C (mm) | 1.45 ± 0.069  502 ± 17  0.171 ± 0.023  130 ± 16  2.89 ± 0.11  1.30 ± 0.053  4.37 ± 0.12 | 1.45 ± 0.071  485 ± 24  0.158 ± 0.016  118 ± 11  3.00 ± 0.12  1.36 ± 0.063  4.51 ± 0.15 | 0.9764  0.1152  0.2543  0.1090  0.0862  0.1062  0.0759 |
| ***Diaphysis*** |  |  |  |
| TOT_CNT (mg/mm)  TOT_DEN (mg/cm³) TOT_A (mm²)  CRT_CNT (mg/mm)  CRT_DEN (mg/cm³)  CRT_A (mm²)  CRT_THK (mm)  PERI_C (mm)  ENDO_C (mm) | 1.23 ± 0.053  662 ± 24  1.85 ± 0.044  1.12 ± 0.055  1046 ± 28  1.07 ± 0.034  0.268 ± 0.0080  4.82 ± 0.055  3.14 ± 0.057 | 1.25 ± 0.057  653 ± 11  1.92 ± 0.067  1.15 ± 0.058  1039 ± 12  1.10 ± 0.048  0.272 ± 0.0082  4.91 ± 0.085  3.20 ± 0.042 | 0.4003  0.4001  ***0.0292***  0.3278  0.5546  0.1231  0.4619  ***0.0304***  ***0.0292*** |

Values are mean ± SD. n=number of individuals.
