# Supplementary material for: Increased Bone Mass in Female Mice Lacking Mast Cell Chymase
Source: PLoS One. 2016 Dec 9;11(12):e0167964. doi: 10.1371/journal.pone.0167964 (PMC5148084; doi:10.1371/journal.pone.0167964)
Supplement: S2 Table — (DOCX) [file pone.0167964.s007.docx]

S2 Table. Femur pQCT characteristics of female mice.

|  | **WT 7 mo**  n=6 | **Mcpt4-/- 7 mo**  n=5 | **p-value** |
| --- | --- | --- | --- |
| Femur length (mm) | 15.7 ± 0.39 | 16.1 ± 0.42 | 0.1569 |
| ***Distal metaphysis*** |  |  |  |
| TOT_CNT (mg/mm)  TOT_DEN (mg/cm³)  TRAB_CNT (mg/mm)  TRAB_DEN (mg/cm³)  TOT_A (mm²)  TRAB_A (mm²)  ENDO_C (mm) | 1.55 ± 0.074  515 ± 31  0.182 ± 0.0075  134 ± 7.6  3.01 ± 0.16  1.36 ± 0.073  4.31 ± 0.23 | 1.58 ± 0.14  449 ± 37  0.180 ± 0.032  112 ± 17  3.52 ± 0.20  1.59 ± 0.091  4.94 ± 0.23 | 0.6421  ***0.0106***  0.9024  ***0.0169***  ***0.00096***  ***0.0012***  ***0.0014*** |
| ***Diaphysis*** |  |  |  |
| TOT_CNT (mg/mm)  TOT_DEN (mg/cm³) TOT_A (mm²)  CRT_CNT (mg/mm)  CRT_DEN (mg/cm³)  CRT_A (mm²)  CRT_THK (mm)  PERI_C (mm)  ENDO_C (mm) | 1.51 ± 0.010  699 ± 37  2.16 ± 0.054  1.39 ± 0.095  1023 ± 40  1.35 ± 0.049  0.32 ± 0.014  5.20 ± 0.067  3.17 ± 0.12 | 1.57 ± 0.055  669 ± 6.8  2.34 ± 0.080  1.43 ± 0.058  1007 ± 18  1.42 ± 0.058  0.32 ± 0.0052  5.42 ± 0.090  3.40 ± 0.070 | 0.2506  0.1056  ***0.0013***  0.3972  0.4110  ***0.0415***  0.8038  ***0.0012***  ***0.0035*** |

Values are mean ± SD. n=number of individuals.
