# Supplementary material for: Increased Bone Mass in Female Mice Lacking Mast Cell Chymase
Source: PLoS One. 2016 Dec 9;11(12):e0167964. doi: 10.1371/journal.pone.0167964 (PMC5148084; doi:10.1371/journal.pone.0167964)
Supplement: S3 Table — (DOCX) [file pone.0167964.s008.docx]

S3 Table. Femur pQCT characteristics of female mice.

|  | **WT 12 mo**  n=4 | **Mcpt4-/- 12 mo**  n=9 | **p-value** |
| --- | --- | --- | --- |
| Femur length (mm) | 15.6 ± 0.26 | 15.7 ± 0.54 | 0.6865 |
| ***Distal metaphysis*** |  |  |  |
| TOT_CNT (mg/mm)  TOT_DEN (mg/cm³)  TRAB_CNT (mg/mm)  TRAB_DEN (mg/cm³)  TOT_A (mm²)  TRAB_A (mm²)  ENDO_C (mm) | 1.43 ± 0.11  458 ± 46  0.168 ± 0.022  120 ± 18  3.12 ± 0.095  1.41 ± 0.045  5.34 ± 0.21 | 1.53 ± 0.099  462 ± 39  0.187 ± 0.028  125 ± 21  3.32 ± 0.22  1.50 ± 0.10  5.49 ± 0.30 | 0.1276  0.8734  0.2583  0.6424  0.1170  0.1212  0.3934 |
| ***Diaphysis*** |  |  |  |
| TOT_CNT (mg/mm)  TOT_DEN (mg/cm³) TOT_A (mm²)  CRT_CNT (mg/mm)  CRT_DEN (mg/cm³)  CRT_A (mm²)  CRT_THK (mm)  PERI_C (mm)  ENDO_C (mm) | 1.44 ± 0.047  634 ± 19  2.26 ± 0.14  1.07 ± 0.033  1178 ± 8.4  0.908 ± 0.025  0.192 ± 0.0048  5.33 ± 0.16  4.13 ± 0.18 | 1.60 ± 0.088  613 ± 24  2.60 ± 0.12  1.16 ± 0.092  1173 ± 26  0.987 ± 0.070  0.193 ± 0.012  5.72 ± 0.13  4.51 ± 0.12 | ***0.0061***  0.1547  ***0.00074***  0.0896  0.7119  0.0531  0.8694  ***0.00068***  ***0.00088*** |

Values are mean ± SD. n=number of individuals.
