# Supplementary material for: Increased Bone Mass in Female Mice Lacking Mast Cell Chymase
Source: PLoS One. 2016 Dec 9;11(12):e0167964. doi: 10.1371/journal.pone.0167964 (PMC5148084; doi:10.1371/journal.pone.0167964)
Supplement: S4 Table — (DOCX) [file pone.0167964.s009.docx]

S4 Table. Femur micro CT characteristics of female mice.

|  | **WT 7 mo**  n=4 | **Mcpt4-/- 7 mo**  n=4 | **p-value** |
| --- | --- | --- | --- |
| ***Distal metaphysis*** |  |  |  |
| Bone volume (mm^3^)  Bone volume/tissue volume (%)  Bone surface (mm^2^)  Bone surface/bone volume (mm^-1^)  Trabecular number (mm^-1^)  Trabecular thickness (mm)  Trabecular separation (mm)  Trabecular bone pattern factor (mm^-1^)  Degree of anisotrophy (range 0-1)  Structural model index | 0.0479 ± 0.023  2.15 ± 1.0  4.01 ± 1.7  85.9 ± 6.0  0.415 ± 0.19  0.0517 ± 0.0025  0.435 ± 0.031  39.9 ± 6.7  1.89 ± 0.53  2.83 ± 0.27 | 0.126 ± 0.039  4.73 ± 1.4  8.28 ± 3.2  64.7 ± 8.2  0.765 ± 0.30  0.0641 ± 0.012  0.4764 ± 0.15  26.1 ± 1.1  1.79 ± 0.37  2.52 ± 0.33 | ***0.01982***  ***0.03630***  0.06639  ***0.01060***  0.1158  0.09786  0.6085  ***0.01812***  0.7776  0.2357 |
| ***Diaphysis*** |  |  |  |
| Bone volume (mm^3^)  Bone surface (mm^2^)  Bone surface/bone volume (mm^-1^)  Total porosity (%) | 0.235 ± 0.0092  4.20 ± 0.11  17.9 ± 0.54  3.30 ± 1.5 | 0.250 ± 0.013  4.44 ± 0.11  17.8 ± 0.61  7.71 ± 2.2 | 0.09940  ***0.02336***  0.7346  ***0.01743*** |

Values are mean ± SD. n=number of individuals.
